# Hippocampal neurogenesis facilitates cognitive flexibility in a fear discrimination task

**DOI:** 10.3389/fnbeh.2023.1331928

**Published:** 2024-01-11

**Authors:** Alonso Martínez-Canabal, Grecia López-Oropeza, Francisco Sotres-Bayón

**Affiliations:** ^1^Department of Cell Biology, Faculty of Sciences, National Autonomous University of Mexico (UNAM), México City, Mexico; ^2^Cell Physiology Institute - Neuroscience, National Autonomous University of Mexico (UNAM), México City, Mexico; ^3^Graduate Program in Biological Sciences, National Autonomous University of Mexico (UNAM), México City, Mexico

**Keywords:** neuroplasticity, newborn neurons, environmental enrichment, fear conditioning, prefrontal cortex, lateral habenula

## Abstract

Hippocampal neurogenesis, the continuous creation of new neurons in the adult brain, influences memory, regulates the expression of defensive responses to threat (fear), and cognitive processes like pattern separation and behavioral flexibility. One hypothesis proposes that neurogenesis promotes cognitive flexibility by degrading established memories and promoting relearning. Yet, empirical evidence on its role in fear discrimination tasks is scarce. In this study, male rats were initially trained to differentiate between two similar environments, one associated with a threat. Subsequently, we enhanced neurogenesis through environmental enrichment and memantine treatments. We then reversed the emotional valence of these contexts. In both cases, neurogenesis improved the rats’ ability to relearn the aversive context. Interestingly, we observed increased hippocampal activity, and decreased activity in the prelimbic cortex and lateral habenula, while the infralimbic cortex remained unchanged, suggesting neurogenesis-induced plasticity changes in this brain network. Moreover, when we pharmacologically inhibited the increased neurogenesis with Methotrexate, rats struggled to relearn context discrimination, confirming the crucial role of neurogenesis in this cognitive process. Overall, our findings highlight neurogenesis’s capacity to facilitate changes in fear discrimination and emphasize the involvement of a prefrontal-hippocampal-habenula mechanism in this process. This study emphasizes the intricate relationship between hippocampal neurogenesis, cognitive flexibility, and the modulation of fear-related memories.

## 1 Introduction

A critical ability for survival and mental health is the capacity of individuals to differentiate between aversive and safe conditions in a changing environment. This capacity, known as cognitive flexibility, involves the dynamic adjustment of responses to ever-changing rules governing these distinctions (Rende, 2000). Cognitive flexibility is closely associated with the highly plastic prefrontal cortex (Young and Shapiro, 2011), a brain region responsible for higher-order cognitive functions. Additionally, the hippocampus, a brain structure renowned for its role in memory acquisition and temporary memory storage (Anagnostaras et al., 1999; Kitamura et al., 2017), contributes significantly to cognitive flexibility through the acquisition and processing of spatial and contextual memories (Epp et al., 2016).

A noteworthy characteristic of the hippocampus is its continuous generation of newborn neurons during adulthood, providing it with remarkable plasticity. These newborn neurons have been associated with pattern separation, the precise discrimination between two similar contexts (Creer et al., 2010; Sahay et al., 2011). Their plastic properties, coupled with the hippocampal-dentate gyrus structure, enable this essential function (Aimone et al., 2011). Additionally, newborn neurons contribute significantly to memory consolidation, regulating the temporal dependence of memories on the hippocampus (Kitamura et al., 2009). They also participate in the clearance of existing hippocampal memories by integrating them into established circuits (Toni et al., 2008; Akers et al., 2014), allowing for the formation of new memories without interference (Epp et al., 2016). Recent research has even revealed that an increased rate of newborn neuron proliferation in the hippocampus can promote the persistence of extinction memories, preventing the resurgence of fear (Martínez-Canabal et al., 2019).

Previous studies have primarily investigated the roles of newborn neurons in memory clearance and consolidation using a single context for training and memory tests (Kitamura et al., 2009; Akers et al., 2014). Experiments exploring pattern separation associated with newborn neurons have focused on modifying neurogenesis before training, leaving gaps in our understanding of post-training neurogenesis’s influence on memory structure (Creer et al., 2010; Sahay et al., 2011). As a result, the extent to which newborn neurons can impact well-established discriminatory memories, facilitate the differentiation of emotional valence between two similar yet distinct contexts (Wang et al., 2009), or aid in rule-switching remains unclear.

Therefore, this study aims to bridge this knowledge gap by investigating whether an increase in neurogenesis, induced over a month through environmental enrichment and memantine administration, can enhance cognitive flexibility in the context of a fear-related task. Specifically, rats were trained to associate one context with an aversive stimulus and another with safety. Subsequently, the rats were challenged to reverse these associations, exposing the previously safe context to an aversive stimulus while maintaining the formerly aversive one as safe. We hypothesized that the increase in adult-born neurons causally regulates rats’ capacity to adapt to this rule-changing paradigm following their initial training. As previously described in the context of the persistence of extinction memory, we found that this enhancement in cognitive flexibility within a fear conditioning paradigm is associated with changes in activity in specific regions of the hippocampus, prefrontal cortex, and habenula (Martínez-Canabal et al., 2019). Our study emphasizes the intricate relationship between hippocampal neurogenesis, cognitive flexibility, and the modulation of fear-related memories.

## 2 Materials and methods

### 2.1 Animals

Male Wistar rats (250 g) were obtained from the breeding colony at the Instituto de Fisiología Celular. They were housed in standard acrylic cages with 2–3 rats per cage, maintained under controlled environmental conditions, including a 12:12 light/dark cycle, regulated temperature, and humidity. The rats had *ad libitum* access to both food and water throughout the experimental procedures. All experimental protocols were conducted with approval from the Institutional Animal Care and Use Committee at the National Autonomous University of Mexico’s Cellular Physiology Institute (CICUAL: FSB35-14).

### 2.2 Contextual discrimination training

In the initial phase of contextual discrimination trials, rats underwent ten daily sessions of contextual fear conditioning (CFC) training lasting 10 minutes each day. Context “A” served as the conditioning environment, characterized by shock grids, a white house light, clear plastic walls, and continuous white noise. During the fifth minute of CFC training, a single 0.8 mA foot shock was delivered. Following an eight-hour interval, the rats were placed in context “B,” a distinct environment without foot shocks (unconditioned context), for a duration of 10 minutes. Context “B” featured triangular dimensions, a shock panel, a red house light, two clear plastic walls, and two white plastic walls. After 9 days of training, on day 10 (Test 1), the rats were assessed for their ability to discriminate between memory contexts by testing them during 10 min in both contexts’ “A” and “B.” In between sessions, all shock grids, panels, and floor trays were cleaned with soap and water, and the chamber walls were wiped clean with wet paper towels.

### 2.3 Reversal training

After a month of neurogenesis manipulation (see below), on day 41 (Test 2), rats underwent a ten-minutes-probe trial in both contexts to assess memory persistence. After the Test 2, rats underwent a reversal contextual fear conditioning (CFC) training. This entailed reassigning the previously conditioned context A as the new safe environment, with the previous unconditioned context B becoming the newly conditioned one. The reversal training maintained the original contextual conditions, shock intensity, and time allocation for each context. On day 50 (Test 3), rats were reintroduced to both contexts, during 10 min in each one, to assess their memory retrieval for this modified paradigm.

### 2.4 Neurogenesis manipulations

#### 2.4.1 Environmental enrichment

We used a well-established environmental enrichment protocol aimed at enhancing adult hippocampal neurogenesis (van Praag et al., 1999a). Following the initial discrimination contextual fear conditioning (CFC) training, rats were divided into two groups: the enriched environment group (EE), which had access to a free-running wheel, an opaque plastic tunnel, and chewable wooden materials, and the non-enriched environment group (non-EE), which were housed in standard acrylic cages.

#### 2.4.2 Memantine treatment

Rats were administered memantine (Mem), a commonly utilized pharmacological approach to stimulate hippocampal neurogenesis (Akers et al., 2014). After the initial memory test, rats received intraperitoneal injections of Mem at a dosage of 25 mg/kg dissolved in saline solution once a week for 1 month, while the control group received saline solution injections (Sal).

#### 2.4.3 Methotrexate treatment

Rats were administered three weekly injections of Methotrexate (Mtx) to suppress hippocampal neurogenesis (Winocur et al., 2016). Following the initial memory test, intraperitoneal injections of Mtx at a dosage of 37.5 mg/kg dissolved in saline solution, in addition to memantine treatment, were administered to this group, while the control group received Mem injections dissolved in saline solution.

### 2.5 Immunohistochemistry

To assess newborn neurons (DCX+) and neuronal activity (c-Fos), we collected brains 90 min after the final memory test. The brains were fixed with 4% paraformaldehyde for 48 h, cryoprotected in a 30% sucrose solution, and sliced into 50 μm sections at-20°C using a Cryostat (Leica, CM 1520) with 300 μm spacing. These brain slices were preserved in an antifreeze solution (40% glycerol, 10% ethylene glycol in PBS) and mounted on gelatine-coated slides. After an alcohol gradient treatment, brain slices underwent antigen retrieval in a citrate buffer solution and a peroxidase-blocking protocol using 3% H2O2 (Sigma). Tissue was then blocked with a solution containing 1% Bovine Serum Albumin and 1% Normal Goat Serum (Jackson Inmunoresearch) in TBS-T for 60 min. Following blocking, brain slices were incubated with primary antibodies (anti-c-Fos1:2500 ab-5, or anti-DCX Cell Signaling #4604S 1:4000) for 48 h at 25°C. Afterward, they were incubated with a secondary antibody 1:1000 (biotinylated goat-anti rabbit, Jackson Inmunoresearch), amplified with kit ABC (elite VECTASAN^®^, Vector). Finally, they were visualized with DAB-Ni in the presence of 0.25% H2O2 (Sigma). Slices were mounted and counterstained with methyl green (Sigma) for nuclear visualization.

### 2.6 Cell counting and analysis

Cell quantification involved manual counting of DCX+ neurons in a Nikon Eclipse at 10× (N.A 0.3). Images of DG brain sections, representing all the structure, were taken at 10× magnification for area measuring (QCapture software v. 7.05) and calculating density of newborn neurons per DG using ImageJ^®^ software.

For c-Fos + nuclei density, images from Cornu ammonis 3 (CA3), Prelimbic cortex (PRL), Infralimbic cortex (IL), Lateral habenula (LHb), and basolateral amygdala (BLA) regions were captured at 10× magnification (N.A 0.3). Automated cell counting in ImageJ was performed, considering criteria of an area between 6 to 60 μm and circularity from 0.7 to 1. And calculated the density calculating nuclei per area. To directly compare c-Fos immunoreactivity in brain regions with different baselines, the expression of c-Fos was evaluated as a normalized contrast of c-Fos immunoreactive cells in treated rats (Mem) relative to its control group (Sal) [*z*-score; as in Martínez-Canabal et al. (2019)]. Z-score denotes salience of activity of a brain structure as described by Wheeler et al. (2013). The z-score represents a standardized value, quantifying the experimental group’s deviation from the mean of the control group in terms of standard deviations. A value of ±1.96 is conventionally deemed significant in this context (*p* < 0.05).

### 2.7 Statistical analysis

Graphs were plotted using Graphpad Prism 8.4. Data were analyzed using two-way ANOVA (with repeated measures when dependent variables were measured at multiple within-subject levels), and significant interactions were decomposed using simple main effects and Bonferroni-corrected pairwise comparisons. Statistical analyses, such as unpaired Student’s two-tailed *t*-tests, repeated-measures analysis of variance with Bonferroni post-hoc comparisons, and Pearson correlation coefficient, were applied to all animal groups using Statistica64^©^ (version 12). The Analysis of Variance and the *t*-tests were performed only after the control group of each experiment showed both normality and variance homogeneity in the Kolmogorov–Smirnov and Levene’s tests. The discrimination ratio was calculated as follows: (% Freezing Ctx. A – % Freezing Ctx. B)/(% Freezing Ctx. A + %Freezing Ctx. B) for the tests 1 and 2 and the formula (% Freezing Ctx. B – % Freezing Ctx. A)/(% Freezing Ctx. A + % Freezing Ctx. B) for test 3 when the aversive contexts switch (Wang et al., 2009).

## 3 Results

### 3.1 Environmental enrichment enhances neurogenesis and discrimination in a cognitive fear flexibility paradigm

**Figure 1:**
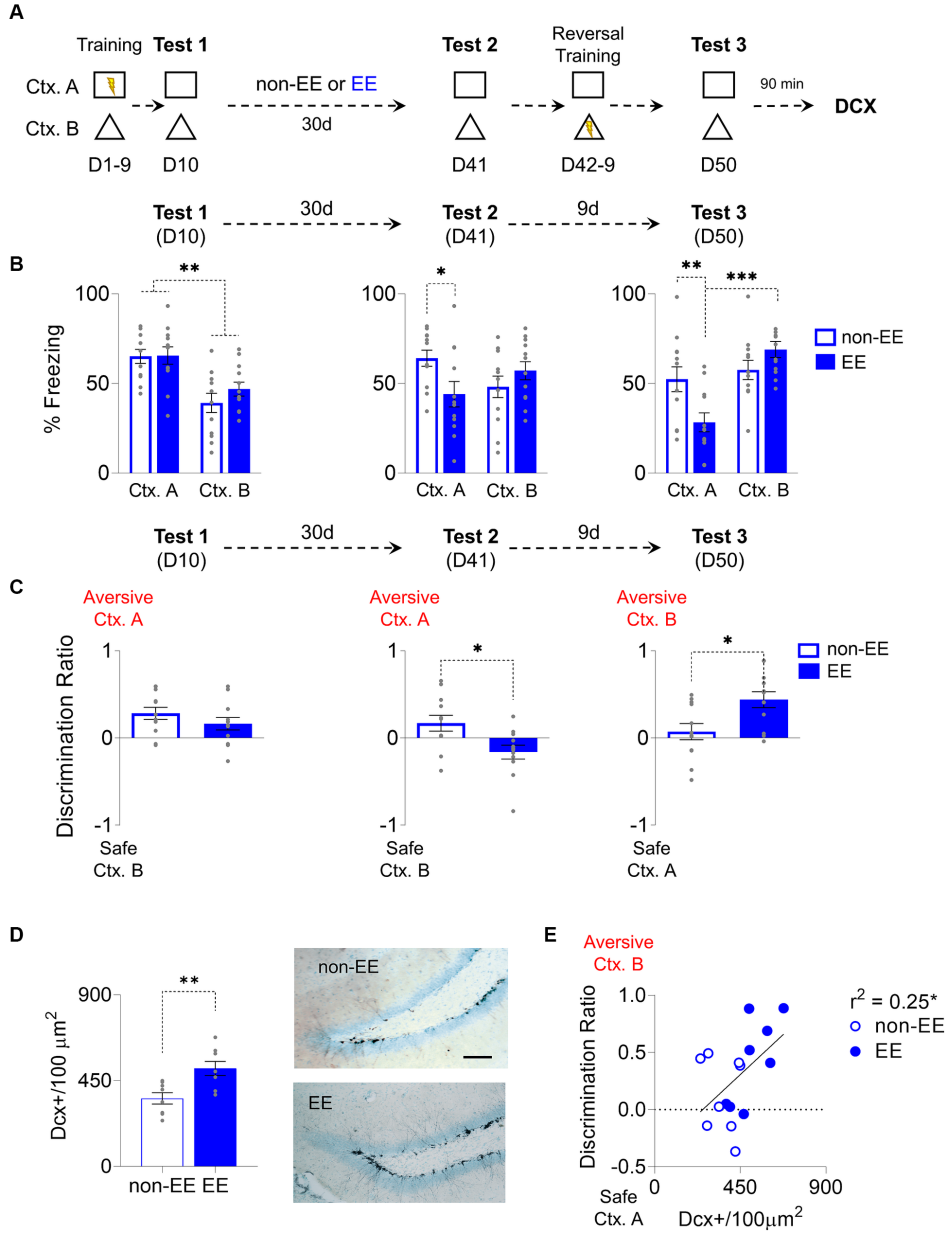
Enriched Environment Enhances Adult Hippocampal Neurogenesis and Improves Cognitive Fear Flexibility. **(A)** For 9 days, rats were trained to distinguish between an aversive and a safe context by associating context A (Ctx. A) with foot shocks and a shock-free context B (Ctx. B). The next day (D10), rats were placed in both contexts without foot shocks to test for discrimination memory. Then, rats were housed for 30 days either in an enriched environment (EE) or regular housing (non-EE). On D41, rats were tested again for memory retrieval in both contexts. The following 8 days, rats underwent a reversal conditioning training where the previously threat context became safe (Ctx. A), and the safe one became aversive (Ctx. B). Finally, on D50, rats were placed again in the same contexts to test for reversal learning memory. **(B)** *Left*. Rats in EE (*n* = 12) and non-EE (*n* = 12) groups learned to discriminate between threat Ctx. A and safe Ctx. B, in D10. Middle. EE rats decreased discrimination memory between the two contexts when exposed to the same contexts D41. *Right*. EE facilitated reversal learning of conditioned contexts, compared to non-EE rats on the last memory test (D50). **(C)** *Left*. EE and non-EE groups discriminated between contexts in the initial test. *Middle*. EE showed lower, near zero, discrimination in D41, compared to the non-EE group. *Right*. EE facilitated discrimination of contexts after reversal learning, as indicated by increased discrimination to the newly aversive context B compared to near zero discrimination levels in the non-EE group on the final memory test (day 50). **(D)** *Left*. Enrichment for 30 days increased hippocampal neurogenesis, evidenced by higher levels of immature neurons (DCX+) in the EE group compared to the non-EE group. *Right*. Representative immunohistochemically stained DCX+ neurons (black) in the subgranular zone of the dentate gyrus of the hippocampus after EE or non-EE. Scale bar: 50 μm. (non-EE, *n* = 8 rats; EE, *n* = 8 rats) **(E)** EE increased neurogenesis and facilitated contextual discrimination, demonstrated by a correlation between DCX+ neurons and discrimination ratio on the reversal retrieval test day. Error bars indicate SEM, *p < 0.05, **p < 0.01, ***p < 0.001.

A group of rats underwent a nine-day training regimen to distinguish between two contexts: an aversive environment, labeled as “A,” involving foot-shock delivery during each presentation, and a second context, denoted as “B” devoid of aversive elements intended to elicit a conditioned response, specifically freezing (see Figure 1A). Freezing responses increased during contextual fear conditioning (CFC) in context A. On the 10th day (D10), a probe test was conducted without shock to assess the initial formation of fear memory in both contexts. Subsequently, the rats were divided into two groups: one housed in standard conditions (non-EE) and the other in an enriched environment (EE). Both groups showed similar levels of freezing in the retention probe in the aversive context A, which is higher than in the non-aversive context B in both groups (Group: F_1,22_ = 0.66, *p* = 0.42; CTX: F_1, 22_ = 31.85, *p* < 0.001, Bonferroni-corrected pairwise comparison: non-EE Ctx. A vs. Ctx. B *p* < 0.001, EE Ctx. A vs. Ctx. B *p* < 0.006,; Group x CTX: F_1,22_ = 0.83, *p* = 0.37) (Figure 1B). Rats were exposed to an enriched environment for 30 days, and both groups underwent a post-enrichment retention probe on day 41 (D41). In this probe, the EE group showed a freezing decrease in the aversive context (Group: F_1,22_ = 0.93, *p* = 0.31; CTX: F_1, 22_ = 0.06, *p* = 0.8; Group x CTX: F_1,22_ = 6.43, *p* = 0.02, Bonferroni-corrected pairwise comparison: non-EE vs. EE Ctx. A *p* = 0.03). After this probe and reversal training, on day 50 (D50), another probe test was conducted. In this case, the EE group learned better the rule change, showing an increase in freezing compared to the non-EE group in context B (Group: F_1,22_ = 1.88, *p* = 0.18; CTX: F_1, 22_ = 12.64, *p* = 0.002; Group x CTX: F_1,22_ = 7,55, *p* = 0.01, Bonferroni-corrected pairwise comparison: non-EE vs. EE Ctx. A *p* = 0.008, EE Ctx. A vs. Ctx. B *p <* 0.001). We used a discrimination ratio, as previously reported by Wang et al. (2009) and Sahay et al. (2011), to evaluate the rats’ capacity to distinguish between the two contexts. This ratio spans from-1, reflecting exclusive freezing behavior in response to Ctx. B, to 1, indicating exclusive freezing behavior in response to context A. A value of 0 indicates an absence of discrimination between the contexts (Figure 1C). In the first probe (D10), both groups recognized the aversive context from the safe one at similar levels (t_22_ = 1.98, *p* = 0.24). However, during the second probe (D41), the non-EE group displayed higher discrimination towards the correct context. (t_22_ = 2.57, *p* = 0.01). Conversely, in the third probe (D50), the EE group showed higher discrimination towards Ctx. B once this became aversive (t_22_ = 2.32, *p* = 0.04).

Chronic environmental enrichment consistently augments neurogenesis (van Praag et al., 1999b). To assess this effect, we conducted immunostaining and quantified immature neurons in the dentate gyrus of the hippocampus. The EE group displayed a notable increase in the density of immunoreactive somas marked by doublecortin (DCX) (t_14_ = 3.32, *p* = 0.005) (Figure 1D). Notably, a modest yet statistically significant correlation emerged between neurogenesis levels and context discrimination ability; heightened neurogenesis levels correlated with enhanced discrimination ratio; the more neurogenesis the higher is the discrimination towards the currently aversive context (*r*^2^ = 0.25, *p* = 0.04) (Figure 1E).

### 3.2 Memantine increases neurogenesis, enhancing cognitive fear flexibility by modifying structure recruitment

**Figure 2:**
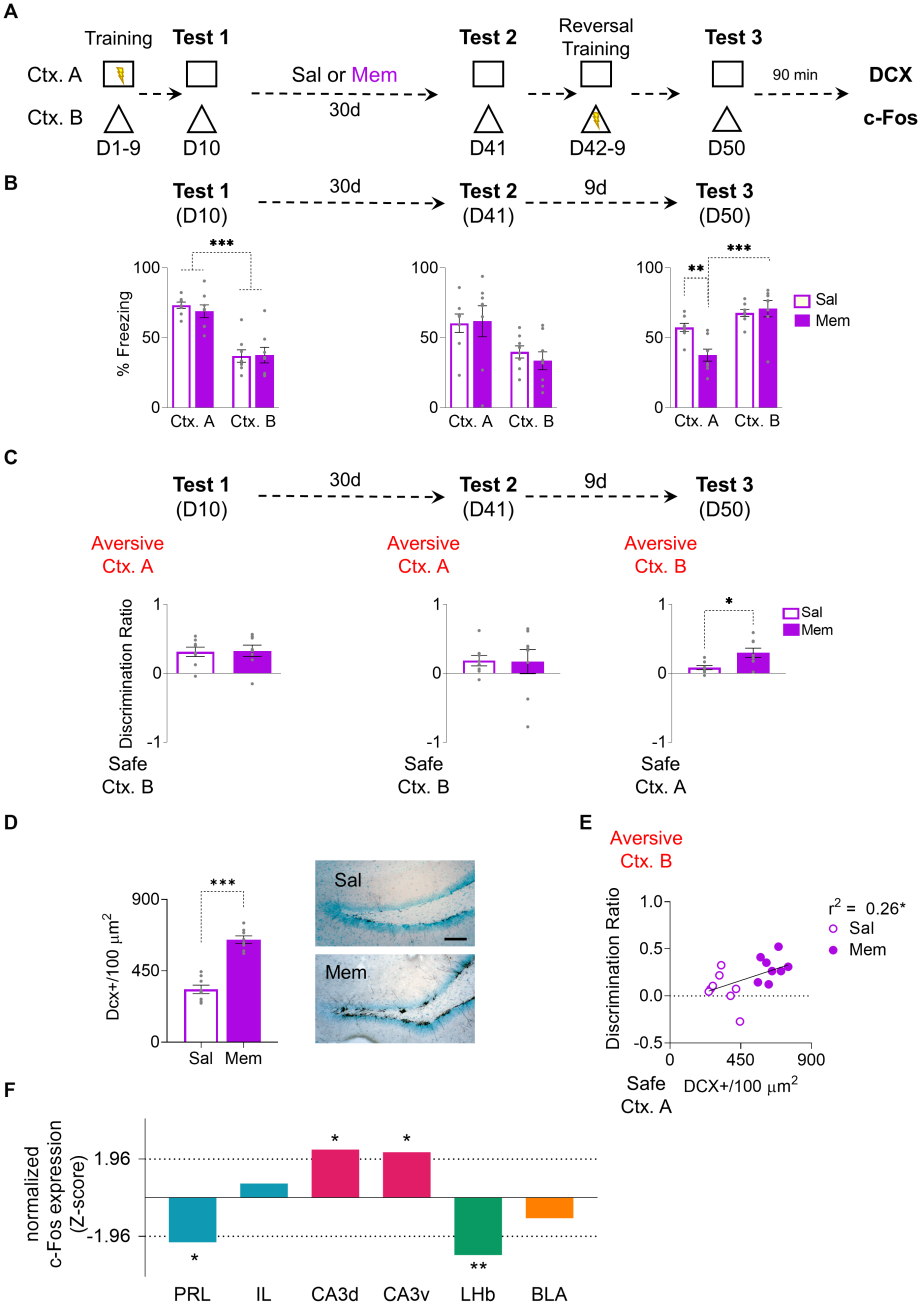
Enhancing Adult Hippocampal Neurogenesis with Memantine Injections Improves Cognitive Fear Flexibility. **(A)** For 9 days, rats were trained to distinguish between threat and safe contexts by associating context A (Ctx. A) with foot shocks while context B (Ctx. B) was shock-free. The next day (day 10), rats were placed in both contexts without foot shocks to test for discrimination memory. Subsequently, the rats received weekly injections of either memantine or saline for a month. On day 41, their memory retrieval for both contexts were tested. During the following 8 days, the rats underwent a reversal conditioning training, wherein the formerly threatening Ctx. A became safe, and the safe Ctx. B became threatening. Finally, on day 50, the rats were reintroduced to the same contexts individually to assess their memory for reversal learning. **(B)** *Left.* On the 10-day test, the Mem (*n* = 8) and Sal (*n* = 8) showed higher fear responses in the aversive Ctx. A but not in the safe Ctx. B. *Middle.* After At D41 Both Mem and Sal rats displayed comparable freezing responses in both contexts. *Right.* At D50, the Mem group recognized the old safe Ctx. B as the new aversive one, while Sal group froze similarly in both contexts. **(C)** *Left*. At D10 Both Sal and Mem showed discrimination towards the aversive Ctx. A. *Middle*. At D41 Both groups maintained similar discrimination indices towards the aversive Ctx. A. *Right*. At D50 Mem facilitated discrimination towards the new aversive Ctx. B. compared to the Sal group. **(D)** *Left*. Memantine treatment increased hippocampal neurogenesis, showing higher levels of immature neurons [doublecortin positive (DCX+)] in the Mem group. *Right*. Representative immunohistochemically stained DCX+ (black) in the subgranular zone of the dentate gyrus of the hippocampus after Mem or Sal treatment. Scale bar: 50 μm. **(E)** Mem increased neurogenesis and facilitated contextual discrimination, demonstrated by a correlation between DCX+ cells and discrimination ratio on D50. **(F)** Data represent normalized contrast of c-Fos immunoreactive cells in treated rats [Mem, (*n* = 6 rats)] as compared to its control group [Sal, (*n* = 6 rats)]. The hippocampus layers CA3d and CA3v show increase in recruitment compared to controls [positive z-scores that exceeded 1.96; *p* < 0.05 (dotted line)], while the prelimbic cortex (PRL) and lateral habenula (LHb) showed reduced recruitment compared to controls (negative z-scores that exceeded −1.96; *p* < 0.05). Error bars indicate SEM, *p < 0.05, **p < 0.01, and ***p < 0.001.

Memantine, a drug known for its effectiveness in increasing neurogenesis (Maekawa et al., 2009), was chosen for its minimal side effects and consistent results. We decided to use this approach to eliminate the potential confounding effects of environmental enrichment and replicate context preference changes previously observed (Figure 2A). After the probe test, following the initial training, the rats were divided into two groups: A memantine-treated group (Mem) and a vehicle-treated group (Sal). Both groups showed the same freezing levels in the aversive context A which were higher than the levels showed in the context B (Group: F_1, 14_ = 0.19, *p* = 0.66; CTX: F_1, 14_ = 57.08, *p* < 0.001, Bonferroni-corrected pairwise comparison: non-EE Ctx. A vs. Ctx. B *p* < 0.001, EE Ctx. A vs. Ctx. B *p* < 0.001; Group x CTX: F_1, 14_ = 0.30, *p* = 0.59) (Figure 2B). The Mem group received the drug weekly for a month after the training, and the Sal group received the equivalent vehicle (isotonic saline solution) injections. After a month of treatment, both groups exhibited consistent freezing levels in the probe test on context A on D41 (Group: F_1, 14_ = 0.1, *p* = 0.76; CTX: F_1, 14_ = 11.62, *p* = 0.004; Group x CTX: F_1, 14_ = 0.29, *p* = 0.59); however, after 10 days of training with Ctx. A being secure and the Ctx. B aversive, Mem increased the freezing response in Ctx. B while decreasing in Ctx. A, demonstrating successful adaptation to the rule change (Group: F_1, 14_ = 3.73, *p* = 0.07; CTX: F_1, 14_ = 31.84, *p* < 0.001; Group x CTX: F_1, 14_ = 8.72, *p* = 0.01, Bonferroni-corrected pairwise comparison: non-EE vs. EE Ctx. A *p* = 0.004, EE Ctx. A vs. Ctx. B *p* < 0.001) (Figure 2B). Context discrimination was assessed through the discrimination ratio. While no differences were observed between groups on D10 (t_14_ = 0.13, *p* = 0.89) and D41 (t_14_ = 0.07, *p* = 0.94), on D50 the Mem group displayed significantly higher discrimination than the Sal group (t_14_ = 2.94, *p* = 0.01) (Figure 2C). As expected, Mem exhibited a higher number of immature neurons immunoreactive to DCX (t_14_ = 8.83, *p* < 0.001) (Figure 2D). Notably, the number of immature neurons, albeit modestly, correlated significantly with the discrimination ratio (*r*^2^ = 0.26, *p* = 0.04) (Figure 2E).

As the increase of neurogenesis in Mem-treated animals was higher than in enrichment-exposed ones, post-probe test c-Fos’s immunoreactivity in Sal and Mem groups was evaluated to determine the activity levels in different brain structures. The data was normalized to Z-scores for comparison. The Mem group displayed decreased activity in the prelimbic cortex (PRL) (*z* = −2.26, *p* = 0.02). In contrast, no significant activity changes were observed in the infralimbic cortex (IL) (*z* = 0.72, *p* = 0.47). Notably, the CA3 layer of the hippocampus exhibited increased activation in the Mem group, both in dorsal CA3 (dCA3) (*z* = 2.44, *p* = 0.01) and ventral CA3 (vCA3) (*z* = 2.31, *p* = 0.02). The lateral habenula (LHb) showed inactivation in the mem group (*z* = −2.92, *p* = 0.003) consistently with prior research (Martínez-Canabal et al., 2019). The Basolateral Amygdala (BLA) did not show changes between groups (*z* = −1.04, *p* = 0.29) (Figure 2F).

### 3.3 Memantine-induced cognitive flexibility disappears after hippocampal neurogenesis reduction

**Figure 3:**
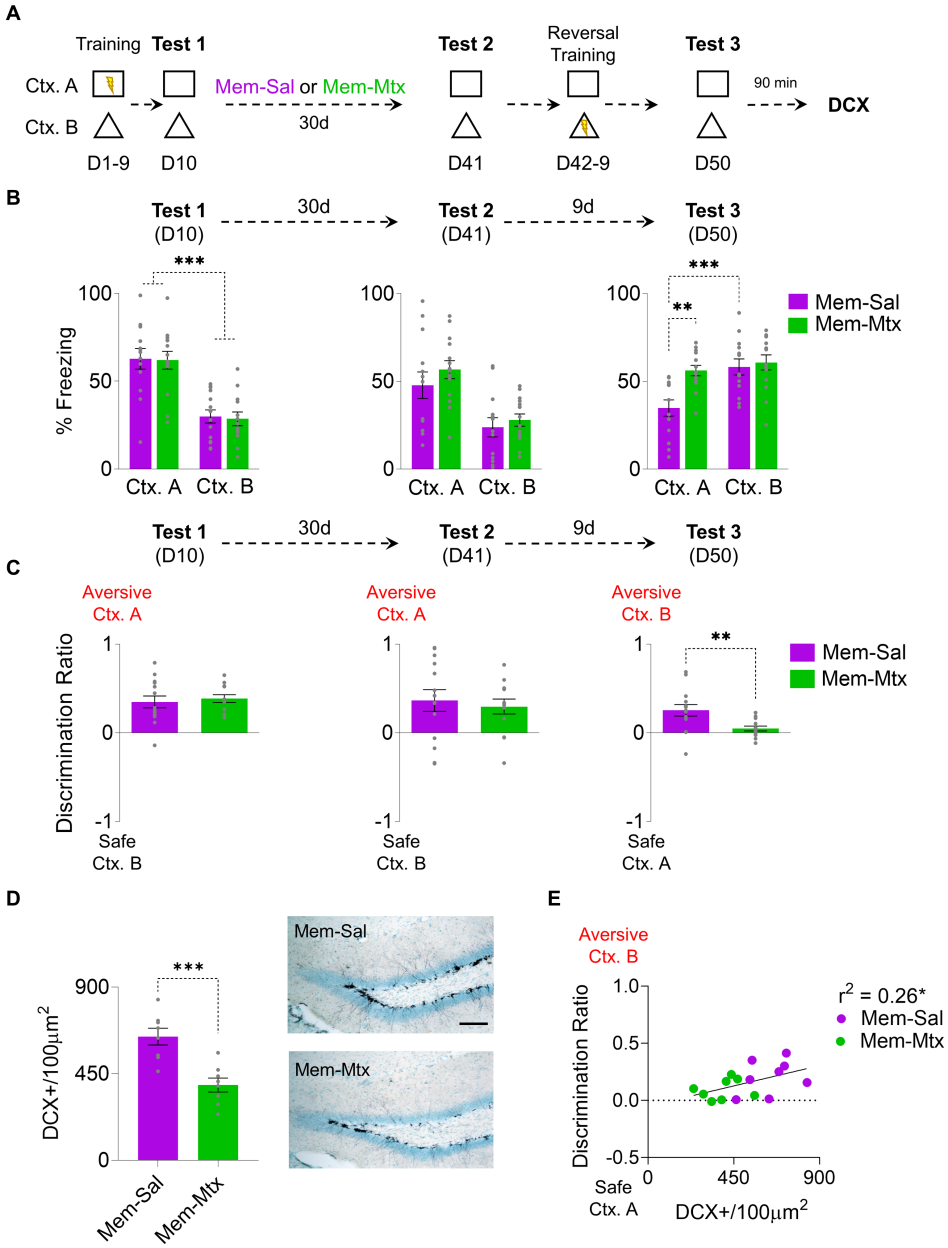
Methotrexate Treatment in Rats with Enhanced Hippocampal Neurogenesis Impairs Cognitive Fear Flexibility. **(A)** For 9 days, rats were trained to distinguish between threat and safe contexts by associating context A (Ctx. A) with foot shocks while context B (Ctx. B) was shock-free. The next day (D10), rats were placed in both contexts without foot shocks to test for discrimination memory. Subsequently, rats received weekly injections of memantine for 1 month and three weekly injections of Methotrexate. On D41, the rats’ memory retrieval for both contexts was reevaluated. Over the next 8 days, the rats underwent a reversal conditioning training, with the previously threatening context A becoming safe and the formerly safe context B becoming threatening. Finally, on D50, the rats were again placed individually in the same contexts to assess their memory for reversal learning. **(B)** *Left*. At D10 Both groups (Mem-Sal = 13; Mem-Mtx = 14) exhibited similar freezing percentages in the aversive Ctx. A in the initial probe trial, higher than in the safe Ctx. B. *Middle.* At D41 after memantine-saline or memantine-methotrexate treatment, both groups showed high freezing levels in Ctx. A. *Right.* During the final probe test at D50, only the Mem-Sal group discriminated between contexts showing higher freezing in the newly aversive Ctx. B and decreased freezing in the newly safe Ctx. A. **(C)** *Left*. At D10 both groups learned to distinguish between contexts, showing discrimination indices toward Ctx. A. *Middle*. At D41 both groups maintained the discrimination. *Right*. At D50 Mem-Sal showed higher discrimination towards the newly aversive Ctx. B, compared to near-zero discrimination levels in the Mem-Mtx group. **(D)** *Left*. Mem-Mtx (*n* = 8 rats) treatment showed neurogenesis ablation compared to the Mem-Sal group (*n* = 8 rats). *Right*. Representative immunohistochemically stained DCX+ (black) in the subgranular zone of the dentate gyrus of the hippocampus after memantine or methotrexate treatment. Scale bar: 50 μm. **(E)** Mem-Sal facilitated contextual discrimination, demonstrated by the correlation between DCX+ cells and the discrimination ratio on the reversal retrieval test day. Error bars indicate SEM, *p < 0.05, **p < 0.01, and ***p < 0.001.

In experiments involving environmental enrichment and memantine, a modest yet statistically significant correlation was observed between the augmentation of newborn neurons and improved discrimination between two contexts with a rule change. To establish causality, it is necessary to ablate neurogenesis to determine if it also blocks the increase of the rule-switching capability generated by neurogenesis. We used the drug methotrexate, which in low dosage is known to be secure without secondary effects and prevents hippocampal proliferation (Winocur et al., 2016; Figure 3A). After the probe’s first test (D10), following the initial training, the rats were divided into two groups: A memantine-saline group (Mem-Sal) and a memantine-methotrexate group (Mem-Met). Both groups showed the same freezing levels in the aversive context A, significantly higher than the safe context B (Group: F_1, 25_ = 0.25, *p* = 0.62; CTX: F_1,25_ = 42.57, *p* < 0.001, Bonferroni-corrected pairwise comparison Mem-Sal Ctx. A vs. Ctx. B *p* < 0.001, Mem-Mtx Ctx. A vs. Ctx. B *p* < 0.001; group x CTX: F_1, 25_ = 0.06, *p* = 0.79). After a month of treatment with both drug combinations, both groups exhibited consistent freezing levels in the probe test on context A on D41 (Group: F_1, 25_ = 1.78, *p* = 0.19; CTX: F_1,25_ = 18.56, *p* < 0.001; Group x CTX: F_1, 25_ = 0.15, *p* = 0.71) (Figure 3B). However, after 10 days of training with the Ctx. A being secure and the Ctx. B aversive, Mem-Sal increased the freezing response in the Ctx. B and decreased the freezing response in the Ctx. A, while Mem-Met kept the same levels of freezing in both contexts (Group: F_1, 25_ = 5.15, *p* = 0.03; CTX: F_1,25_ = 30.03, *p* < 0.001; Group x CTX: F_1, 25_ = 13.58, *p* = 0.001, Bonferroni-corrected pairwise comparison: Mem-Sal vs. Mem-Mtx Ctx. A *p* = 0.001, Mem-Sal Ctx. A vs. Ctx. B *p* < 0.001) (Figure 3B). The preference index showed no differences between groups on D10 and D41; by D50, the Mem-Sal group displayed significantly higher discrimination than the Mem-Met group towards the newly aversive Ctx. B(t_25_ = 4.09, *p* < 0.001) (Figure 3C). Mem-Met group has 40% reduction of DCX immunoreactive cells relative to Mem-Sal group (t_14_ = 4.46, *p* < 0.001) (Figure 3D). Notably, the number of immature neurons, albeit modestly, correlated significantly with the discrimination ratio, the more neurogenesis the higher is the discrimination towards the currently aversive context (*r*^2^ = 0.26, *p* = 0.04) (Figure 3E).

## 4 Discussion

Our study provides evidence that adult hippocampal neurogenesis plays a critical role in enhancing the cognitive flexibility of rats in distinguishing between previously safe and now aversive contexts, particularly when rules are switched. By employing a naturalistic approach involving environmental enrichment and the neurogenesis-enhancing drug memantine, we have demonstrated that increased neurogenesis correlates with the rats’ improved ability to adapt to rule changes in their learned responses. This effect is emphasized by the activation of the CA3 region of the hippocampus, reduced activity in the prelimbic cortex and the lateral habenula, and a decrease in freezing responses, consistent with prior research (Martínez-Canabal et al., 2019; Velazquez-Hernandez and Sotres-Bayon, 2021). Newborn neurons of the dentate gyrus project their axons to the apical layer of CA3, which makes CA3 the hippocampal area more affected by neurogenesis modulation, as was previously reported (Martínez-Canabal et al., 2019) where there is no activity change in the granule cell layer of the dentate gyrus. However, recent studies suggest that downstream activity of the evaluated circuit, including the lateral entorhinal cortex and the granule cell layer of the dentate gyrus, can participate in cognitive flexibility tasks (Yun et al., 2023) which suggests that the hippocampal formation as a whole can be involved in this type of behavioral task. Our findings revealed that neurogenesis is not only relevant in the context of spatial memory but also has a significant impact on the processing of fear-related memories. This finding highlights the interconnectedness of neurogenesis, cognitive flexibility, and the modulation of fear-related memories.

Previous research indicates that enhanced neurogenesis in the hippocampus following initial episodic-like training facilitates memory modulation across various paradigms. This phenomenon is thought to occur due to newborn neurons’ modification of previously established circuits, reducing memory retrieval capabilities while increasing hippocampal plasticity and associated circuitry. Earlier studies have demonstrated reduced fear expression, improved spatial memory (Akers et al., 2014), spontaneous recovery of fear extinction (Martínez-Canabal et al., 2019), and enhanced cognitive flexibility in spatial memory tasks (Epp et al., 2016) as outcomes of increased neurogenesis.

Our present study employed a slow contextual learning paradigm involving one aversive and one safe context. These contexts switched after a month, a timeframe generally considered sufficient for the proliferation and maturation of newborn neurons (Ambrogini et al., 2004). Considering previous literature, we expected that the behavior *per se* did not generate any change in neurogenesis expression and the control groups should be considered to be in the regular neurogenesis baseline (Lerch et al., 2011). We anticipated that the retention probe following neurogenesis enhancement would not yield significant group differences due to memory consolidation during the ten-day training period when engrams can maturate outside the hippocampus (Kitamura et al., 2017). As expected, the two memantine experiments did not reveal any notable differences between groups; however, the environmental enrichment experiment showed decreased memory retention. The reduction in the correct discrimination in the environmental enriched group reflects a reduction in memory retention. This memory expression decrease is unlikely due to neurogenesis interference because the increase in neurogenesis occurs after the initial memory consolidation period, and the decay of hippocampal dependence on memory already started. This effect could be attributed to nonspecific effects of environmental enrichment and aerobic exercise, as neurotrophins like BDNF increase during enrichment and can modulate memory circuits independently (Peters et al., 2010); also aerobic exercise increases the expression of other critical memory modulatory molecules like FGF, TrkB, and CREB (Lee et al., 2014). Despite this, reversal learning among all animals with enhanced neurogenesis remained consistent after the second training phase, occurring 40 days after the initial training. We designed the addition of an ablation experiment to establish causality, further supporting the assertion that newborn neurons are responsible for the observed cognitive flexibility. We used the drug Methotrexate to block hippocampal proliferation and evaluate the effect over cognitive flexibility caused by increased neurogenesis. In the control groups there was no discrimination between contexts; therefore, we conclude that in the present paradigm only the neurogenesis enhancement generates the increase in discrimination, but an ablation solely would not detect any behavioral effect.

Although the newborn neurons facilitate cognitive flexibility, the precise mechanism remains unclear. It can be attributed to the enhancement of extinction memories (Martínez-Canabal et al., 2019) and an increased pattern separation capability, previously reported in a similar behavioral paradigm (Sahay et al., 2011). The original safe context shows high freezing levels constantly, although always lower than the aversive context; this effect can be attributed to generalization; therefore, the rule change observed in the discrimination ratio during retraining can be more attributed to extinction and pattern separation than a relearning *per se*. In the two experiments with memantine neurogenesis increase, at the beginning of the second phase of training, there was no memory degradation; in this case, cognitive flexibility cannot be attributable to an initial forgetting or incapacity for retrieval; therefore, the cognitive flexibility appears as an isolated emergent consequence of the extended circuit modulation derived from the addition of newborn neurons. The ability to distinguish between the two contexts is attributable to the pattern separation capabilities of the dentate gyrus and CA3 circuits, which some authors suggest is enhanced by the different plastic properties of newborn neurons (Aimone et al., 2011). However, the main effect of cognitive flexibility is attributable to memory circuits’ modulation generated by adding new neurons to the circuits and by recruiting specific extrahippocampal structures. In this research, we evaluated the medial prefrontal cortex, which is the prefrontal area most associated with fear response modulation (Burgos-Robles et al., 2009; Sotres-Bayon et al., 2012). However, other cortical structures, such as the orbitofrontal cortex (OFC) or the anterior cingulate cortex (ACC), are associated with reverse learning and remote memory. The habenula is a structure associated with the emotional valence of distinct environmental factors (Proulx et al., 2014) but is also associated with decision-making (Baker et al., 2015) and fear modulation (Velazquez-Hernandez and Sotres-Bayon, 2021). In the present study, the habenula decreased activity when the rats were exposed to the formerly aversive context, possibly due to increased neurogenesis. If that were the case, the enhanced hippocampal plasticity generated by newborn neurons may modulate the habenula response to emotional stimuli. However, more studies are needed to determine the causal relationship between habenula activity and neurogenesis, and precise analysis of the circuits underlying the relationship between newborn neurons and habenula would also be important.

The present research demonstrates that the capacity of newborn neurons to influence rule changes in cognitive tasks extends beyond spatial memory tests (Anacker and Hen, 2017; Amelchenko et al., 2023). Notably, our results indicate that the facilitation of rule changes resulting from increased neurogenesis can also be applied to memories associated with fear. This finding confirms that neurogenesis not only enhances extinction processes (Pan et al., 2012; Martínez-Canabal et al., 2019) but also facilitates relearning. Previous experiments show that neurogenesis impacts cognitive flexibility in other memory tasks, therefore, the relearning process can be attributed to memory engrams and not only to fear modulation (Epp et al., 2016). Hippocampal neurogenesis is closely linked to emotional regulation. For instance, chronic use of serotonin selective reuptake inhibitors is known to increase hippocampal neurogenesis and the plasticity associated with new neurons (Santarelli, 2003). Conversely, reduced levels of neurogenesis have been observed in patients with various psychopathological conditions related to emotional regulation, such as major depression disorder and post-traumatic stress disorder (Lucassen et al., 2010). These conditions often coincide with deficits in cognitive flexibility (Moulds et al., 2020). Therefore, this study not only sheds light on the role of neurogenesis in facilitating cognitive flexibility but also contributes valuable insights into the intrinsic mechanisms that could be harnessed for the treatment of neuropsychological disorders.

While this report underscores the importance of neurogenesis in the context of rule switching in contextual fear learning, a comprehensive understanding of the underlying mechanisms at structural, physiological, and molecular levels requires further exploration. Future studies should investigate memory traces in various brain areas associated with cognitive flexibility and decision-making. Additionally, exploring the circuit mechanisms through which hippocampal plasticity impacts other brain structures is vital, and optogenetic techniques offer promising avenues for uncovering these neural connections. While the present study only uses male rats, expanding the findings to female rats and other animal models is necessary. Previous studies showed that estradiol levels, or estrous cycle stages in rodents, do not affect hippocampal neurogenesis (Lagace et al., 2007); however, although the levels of fear memory are equivalent in both male and female rats, females express fear with higher motor responses (Shanazz et al., 2022). Therefore, there is no straightforward prediction of how female rats would respond to the present behavioral task and how their neurogenesis would affect their responses.

In summary, our findings emphasize the substantial role of neurogenesis in enhancing context discrimination amid changing aversive rules, even at remote time points beyond the typical consolidation periods. This not only highlights the importance of neurogenesis in cognitive flexibility but also holds potential for the development of therapeutic strategies for brain disorders associated with deficits in emotional regulation and cognitive flexibility.

## Data availability statement

The raw data supporting the conclusions of this article will be made available by the authors, without undue reservation.

## Ethics statement

The animal study was approved by Comité Interno para el Cuidado y Uso de Animales de Laboratorio del Instituto de Fisiología Celular, Universidad Nacional Autónoma de México. The study was conducted in accordance with the local legislation and institutional requirements.

## Author contributions

AM-C: Conceptualization, Formal analysis, Funding acquisition, Supervision, Writing – original draft, Writing – review & editing. GL-O: Formal analysis, Investigation, Visualization, Writing – original draft, Writing – review & editing. FS-B: Conceptualization, Formal analysis, Funding acquisition, Methodology, Supervision, Visualization, Writing – original draft, Writing – review & editing.
